# GS4PB: An R Shiny application to facilitate a genomic selection pipeline for plant breeding

**DOI:** 10.1002/tpg2.70150

**Published:** 2025-12-11

**Authors:** Vishnu Ramasubramanian, Cleiton A. Wartha, Lovepreet Singh, Paolo Vitale, Sushan Ru, Siddhi J. Bhusal, Aaron J. Lorenz

**Affiliations:** ^1^ Department of Agronomy and Plant Genetics University of Minnesota Saint Paul Minnesota USA; ^2^ International Maize and Wheat Improvement Center (CIMMYT) Texcoco Mexico; ^3^ Small Fruit Breeding and Genetics, Department of Horticulture Auburn University Auburn Alabama USA

## Abstract

The implementation of genomics‐assisted breeding methodologies is helping to drive the genetic gain required to meet the grand challenge of producing more food using fewer resources in the face of a changing climate. Despite the documented usefulness of genomics‐assisted breeding toward this end, its full infusion into most small‐ and medium‐sized breeding programs is still incomplete. One major reason for limited routine application of genomic selection among most such programs is the lack of a single integrated software tool capable of assisting breeders throughout the entire genomic prediction pipeline. To help address this need, we have implemented a streamlined genomic prediction and selection pipeline designed for plant breeding programs using open‐source tools. The steps implemented in the pipeline include processing genotypic data (e.g., filtering and imputing genotypic data), merging genotypic and phenotypic data, collecting enviromics covariates, estimating environmental kinship, optimizing training sets, cross‐validating genomic prediction models, and implementing genomic prediction for single or multiple traits across single or multiple environments. Herein, we describe an R Shiny web application named “GS4PB” (Genomic Selection For Plant Breeding) that implements the above steps in the pipeline and discuss the rationale for each of the tools in the pipeline. We used this GS4PB application to conduct an experiment comparing phenotypic and genomic selection, and showed genomic selection worked as well as phenotypic selection for advancement of breeding lines. This publicly available analysis tool will help to lower entry barriers into advanced techniques of genomic prediction, enabling breeders to take advantage of these technologies to help drive genetic gain.

## Plain Language Summary

The inclusion of genomic technologies into cultivar development is helping to meet the grand challenge of producing more food using fewer resources in the face of a changing climate. Despite this, the full infusion of genomic selection into plant breeding is still incomplete. To address challenges related to software availability, we have implemented a streamlined genomic prediction and selection pipeline designed for plant breeding programs using open‐source software tools. This publicly available analysis tool will help to lower entry barriers into advanced techniques of genomic prediction, enabling breeders to take advantage of these technologies to help drive genetic gain.

**Abbreviations**
AI: artificial intelligence
AYT: advanced yield trial
BGGE: Bayesian genotype and genotype‐by‐environment
BLUE: best linear unbiased estimate
CV: cross validation
GB: gigabytes
G‐BLUP: genomic best linear unbiased prediction
GS: genomic selection
GS4PB: Genomic Selection For Plant Breeding
G × E: genotype‐by‐environment
HD: high density
LD: low density
LD‐KNNi: linkage disequilibrium *k*‐nearest neighbor imputation
LOTO: leave‐one‐trial‐out
MET: multi‐environment trial
ML: machine learning
MTME: multi‐trait and multi‐environment
PA: predictive ability
PC: principal component
PEV: prediction error variance
PS: phenotypic selection
PYT: preliminary yield trial
SNP: single‐nucleotide polymorphism
TASSEL: Trait Analysis by Association, Evolution and Linkage

## 1 INTRODUCTION

Recent technological progress in the field of plant breeding, including genotyping, high‐throughput phenotyping, in‐depth environmental characterization (“enviromics”), and artificial intelligence (AI), is driving a new wave of innovation in plant breeding. These tools have enabled data‐driven selection processes wherein breeders use extensive datasets to make optimized breeding decisions (Costa‐Neto et al., 2021; Crossa et al., 2024, 2022; Xu, 2016). One of the core components of this new approach is the genomic prediction methodology integrating all three domains to help breeders make selection decisions (Crossa et al., 2024, 2022). Genomic prediction uses genome‐wide molecular marker data to predict the genetic values of breeding lines, supporting breeders in identifying promising lines to advance. Genomic prediction employs a wide range of parametric and nonparametric statistical models to estimate parameters using a training dataset composed of genomic data, phenotypic data, and potentially environmental data characterizing tested and target environments. The estimated model parameters are then used to predict the genetic values of selection candidates (target population) using only genomic information (Endelman, 2011; Heslot et al., 2012; Howard et al., 2014; Lopez‐Cruz et al., 2015; Pérez & de Los Campos, 2014).

Both parametric and nonparametric models have been used in genomic prediction. Common parametric models include a variety of linear mixed models in frequentist and Bayesian frameworks, such as genomic best linear unbiased prediction (G‐BLUP), ridge regression, and the so‐called Bayesian alphabet models (Endelman, 2011; Gianola et al., 2009; Habier et al., 2011; Meuwissen et al., 2001; Whittaker et al., 2000). Nonparametric models include a variety of machine learning (ML) techniques such as support vector machines, random forests, neural networks, and deep learning methods (Gianola et al., 2011; González‐Camacho et al., 2012; Heslot et al., 2012; Howard et al., 2014; Jubair & Domaratzki, 2022; O. A. Montesinos‐López et al., 2022, 2021, 2018).

Multi‐trait and multi‐environment (MTME) models hold the potential to leverage estimated covariances between traits and environments to enhance prediction accuracy, but whether a more complex model results in better accuracies depends upon the strength of genetic covariances and heritability, and thus is specific to the dataset at hand (Costa‐Neto et al., 2021; Granato et al., 2018; Jarquin et al., 2014; Jia & Jannink, 2012; Lado et al., 2018; A. Montesinos‐López et al., 2016, 2018; O. A. Montesinos‐Lopez et al., 2018). Using MTME models, however, comes at the cost of increased model complexity and computational resource requirements.

The capacity to model enviromic data in genomic prediction for incorporating genotype‐by‐environment (G × E) interactions opens the opportunity to make predictions for new environments in which new breeding lines have not yet been tested (Bernardo, 2020; Costa‐Neto et al., 2021; Crossa et al., 2022). This could also involve future environments of different climate change scenarios by incorporating climate change forecast models into the environmental information (Chenu et al., 2009; Crossa et al., 2022; de los Campos et al., 2020).

Genomic prediction and selection have been widely studied and adopted in public and private programs over the last decade. They can be applied throughout the variety development pipeline, including, but not limited to, design of crosses, rapid recurrent selection, early‐generation advancement, and sparse testing strategies (Wartha & Lorenz, 2021). Each potential application is an opportunity to increase the efficiency and effectiveness of a breeding pipeline, with full adoption promising to increase the rate of genetic gain (Gholami et al., 2021).

Scale and success in implementing genomic selection (GS), however, greatly vary from organization to organization, with variation in the degree of adoption largely determined by organizational resources. Many constraints faced by public programs and small‐ to medium‐sized private programs include the cost of genome‐wide genotyping, availability of personnel trained in advanced genomics‐assisted breeding analytics, and rapid turnaround times. These challenges necessitate the development of a streamlined, easy‐to‐use software pipeline to routinely implement GS (Wartha & Lorenz, 2021).

There are numerous interlocking steps in genomic prediction and selection pipelines, such as HD genotyping of parental lines or reference populations, low‐density (LD) genotyping of newly derived breeding progenies, effective management and processing of both phenotypic and genotypic data, enviromics data processing, imputation from LD to HD marker density on the progenies, and model training and calculation of genomic predictions. Such pipelines could also include additional steps, such as training set optimization and cross validation (CV) to improve and assess prediction accuracy. For multi‐environment trials (METs), prediction models also need to consider G × E interaction effects if they are determined to be an important source of variation.

The steps comprising GS pipelines are typically implemented using various R packages that have been created for specific purposes (Costa‐Neto et al., 2021; Crossa et al., 2025; Endelman, 2011; Granato et al., 2018; Pérez‐Rodríguez & de Los Campos, 2022; Xavier et al., 2020). It can be cumbersome to use these packages separately in an arbitrarily chosen computing environment. In addition, most GS programs face the problem of rapid turnaround time during the “advancement season,” typically entailing a very short timeline to gather all the genotypic data, process it, and make predictions to enable selection decisions. This necessitates a streamlined approach to analysis.

Issues that affect the reproducibility of any data analysis project and distribution of scientific software also affect the implementation of a GS pipeline. Several solutions have been developed over the past two decades to address reproducibility issues in scientific computational workflows at large as well as within the specific context of bioinformatics applications. Dependency management, containerization, and continuous integration practices have been proposed as solutions to this issue (Beaulieu‐Jones & Greene, 2017; Boettiger, 2015). Several tools that have become popular, such as Docker containers (Merkel, 2014), are available for streamlined implementation of data analysis applications.

To this end, we have implemented some of the best open‐source tools available for each of the steps in the pipeline and strung together various programs and software applications into a single streamlined Shiny application in R (Chang et al., 2025). Our choice of the Shiny application in the R environment was mostly dictated by the availability of well‐tested and extensively documented packages for genomic prediction in R. In addition, we have used the Docker containers to ease the replication of the computing environment for the user. For facilitating production‐grade deployment, the app is organized into an R package using the golem framework (Fay et al., 2024). The golem framework simplifies automated testing and continuous integration of apps. We hope this app helps to solve some of the issues related to the speed and reproducibility of genomic prediction and selection workflows.

## 2 GENOMIC PREDICTION AND SELECTION PIPELINE DESCRIPTION

**FIGURE 1:**
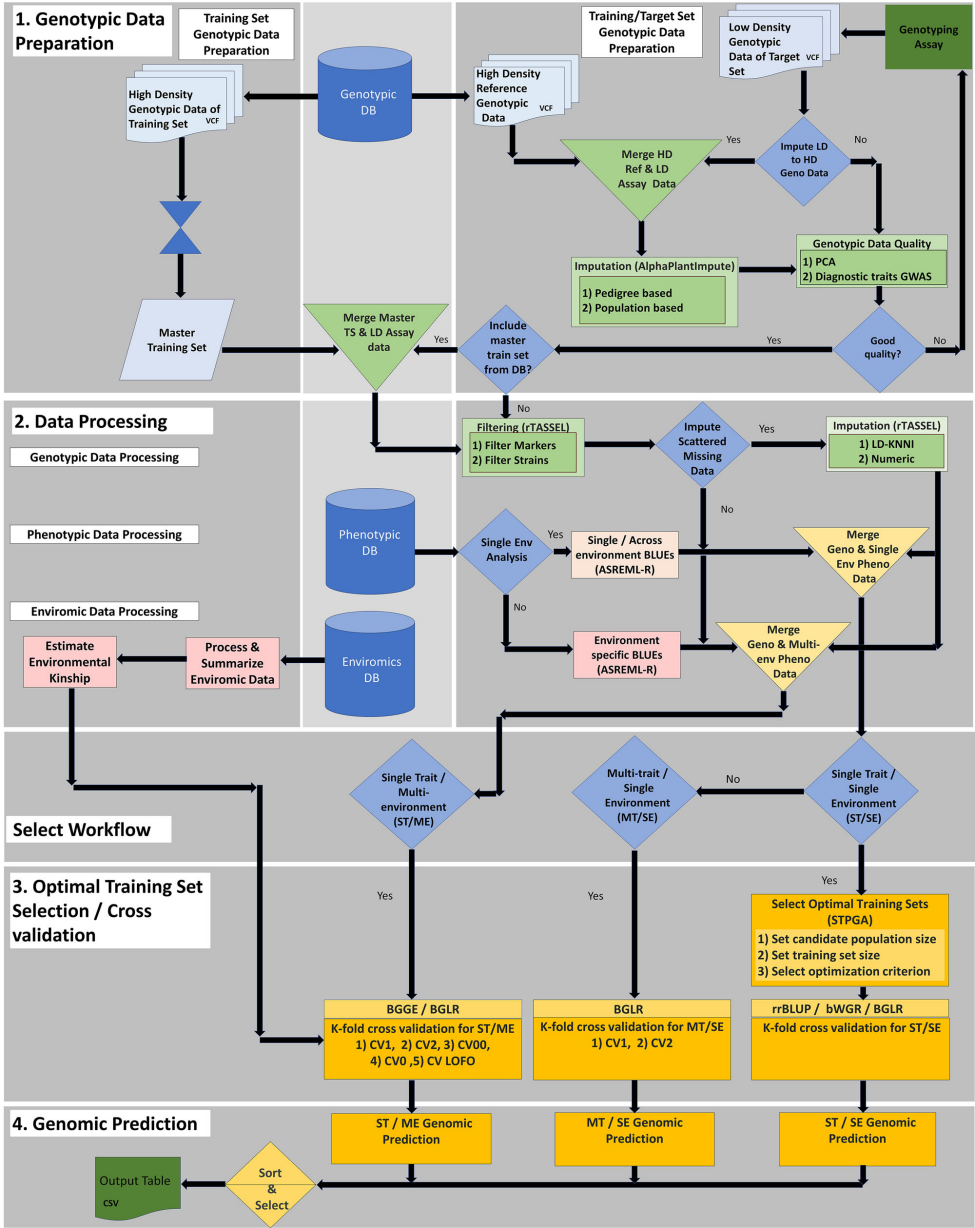
Schematic of a genomic selection pipeline implemented in GS4PB (Genomic Selection For Plant Breeding). The pipeline is split into four steps: (1) genotypic data preparation, (2) data processing, (3) optimal training set selection and cross validation, (4) genomic prediction. (1) High‐density (HD) genotypic data from one or several programs or historical populations are retrieved from databases and compiled into a master training set and filtered to create a high‐quality training dataset. The target population assayed using low‐density (LD) genotypic data is combined with HD genotypic data from parental lines or a reference population for imputation from a LD to an HD marker set using the AlphaPlantImpute program. The imputed data are then filtered to create high‐quality target data, which is then merged with the training data. (2) The data processing step is split into three tracks: (a) genotypic data processing, where the merged genotypic data from Step 1 are filtered for minimum missing frequency for markers, strains, and minor‐allele frequency as well as minimum missing frequency for strains; (b) phenotypic data processing are done externally, where the user can select a single‐/multi‐environmental workflow. For a single‐environment workflow, best linear unbiased estimators (BLUEs) are estimated in one environment or across environments, whereas for a multi‐environmental workflow, BLUEs are estimated within each of the environments. The processed genotype and phenotype data are then merged to create a combined data table. (c) For a multi‐environment workflow, enviromics data can be collected from public databases for the estimation of the environmental kinship matrix. (3) Optimal training set selection and cross validation: An optional training population optimization step is implemented using the *STPGA* package for a single‐trait/single‐environment workflow, followed by a cross validation routine, whether the training population optimization step was elected or not. Cross validation for multi‐trait/single‐environment and single‐trait/multi‐environment analysis can also be performed. (4) Genomic prediction: Genomic predictions are calculated according to a variety of single‐trait, multi‐trait, and multi‐environment models selected by the user. Predictions and the reliability of predictions are output. BGGE, Bayesian genotype and genotype‐by‐environment; BGLR, Bayesian generalized linear regression; bWGR Bayesian whole‐genome regression; CV, cross validation; DB, database; LD‐KNNi, linkage disequilibrium *k*‐nearest neighbor imputation; STPGA, selection of training population using genetic algorithms; TASSEL, Trait Analysis by Association, Evolution and Linkage; TS, training set; VCF, variant call format.

An overview of the GS workflow implemented in the GS4PB (Genomic Selection For Plant Breeding) app is depicted in Figure 1. Each step is described in the following sections.

### 2.1 Data input and quality control

**FIGURE 2:**
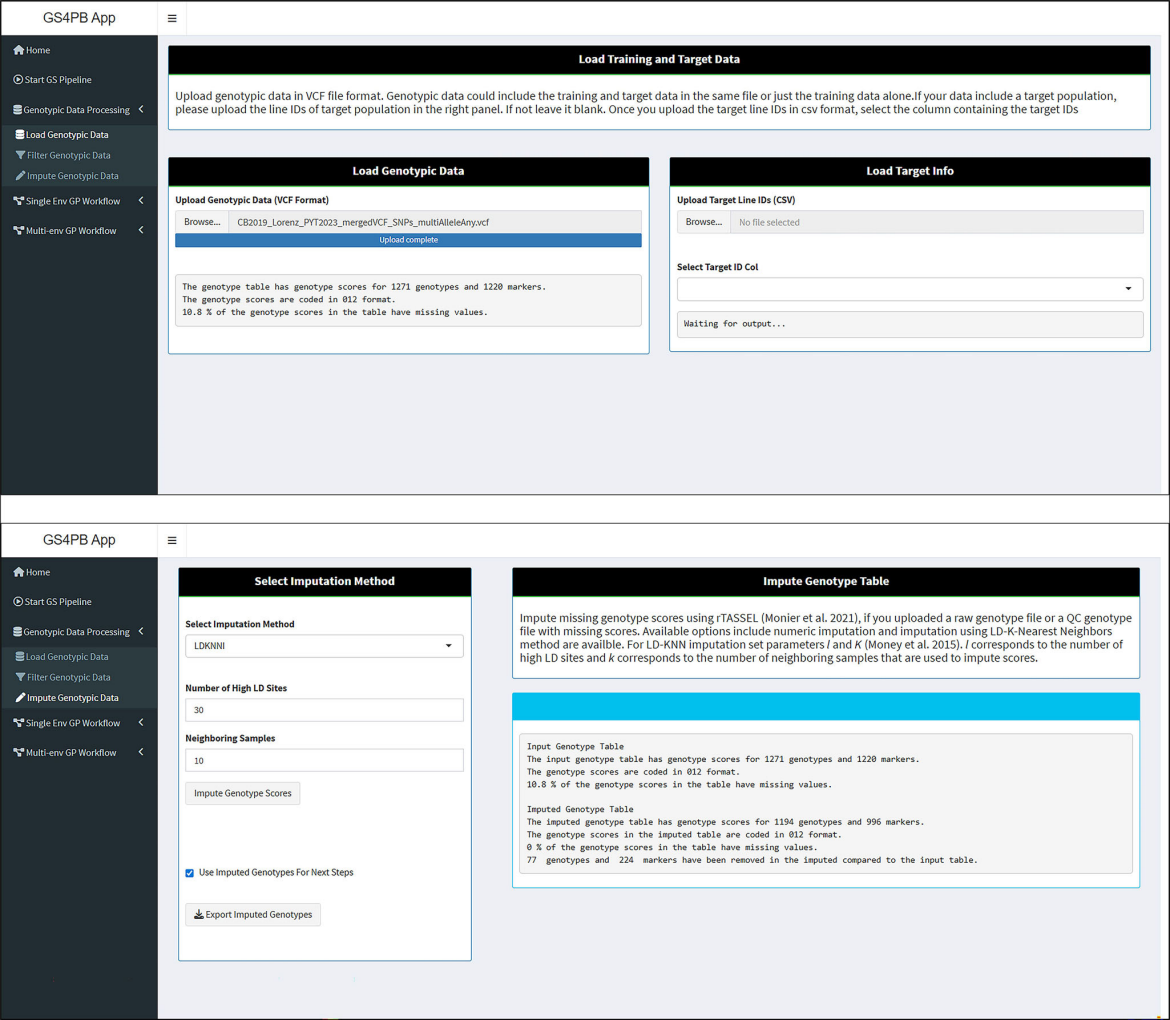
Screenshots of the GS4PB (Genomic Selection For Plant Breeding) app page in which genotypic data is loaded (top panel) and the imputation page (bottom panel). The genotypic data are loaded in a variant call format (VCF). Once loaded, basic statistics like the number of strains and markers are displayed along with the percentage of missing scores. The imputation panel provides options to select the imputation method and set method‐specific parameters. Once the imputation is completed, statistics like the one in the top panel are displayed for both the input and output data tables. CSV, comma‐separated values; GP, genomic prediction; GS, genomic selection; LD, low density; LD‐KNNi, linkage disequilibrium *k*‐nearest neighbor imputation; QC, quality control; TASSEL, Trait Analysis by Association, Evolution and Linkage.

The genotype units most often used in this analysis workflow would likely be breeding lines comprising a crop cultivar breeding pipeline. Because data could also be used from released cultivars or other types of genotypic units such as “progeny rows,” we will refer to the genotypic units generally as “strains” herein. Genotypic data in “variant call format” can be loaded via a user interface (Figure 2). Loaded data are read into a TASSELGenotypePhenotype (where TASSEL stands for Trait Analysis by Association, Evolution and Linkage) object, which is an R S4 object with slots for storing meta information, and genotype and phenotype tables (Monier et al., 2022). This method of data import facilitates subsequent steps, such as filtering and imputation implemented in the *rTASSEL* package (Monier et al., 2022). The functions “filterGenotypeTableSites” and “filterGenotypeTableTaxa” in *rTASSEL* are used for filtering (Monier et al., 2022). Markers can be filtered on the minor‐allele frequency and frequency of missing data, whereas strains (referred to as “taxa” in TASSEL) can be filtered based on the frequency of missing sites.

### 2.2 Genotype imputation

Two methods implemented in rTASSEL, the linkage disequilibrium *k*‐nearest neighbor imputation (LD‐KNNi) method (Money et al., 2015) and numeric imputation, are available within GS4PB to impute missing marker genotype scores (Figure 2). The LD‐KNNi method is a population‐based imputation method with three input parameters: the number of high linkage disequilibrium sites, number of nearest neighboring samples, and the maximum distance in the genome to be considered for linkage disequilibrium estimation between sites. However, for the numeric imputation method, the user can set the number of nearest neighboring samples and a type of distance measure that is set to “Euclidean” as the default. An additional logical input parameter sets whether the imputation is to be performed by estimating the mean value (Monier et al., 2022). By default, a mean value is estimated from this select number of neighboring samples for each of the markers, and the missing values are imputed.

### 2.3 LD to HD genotype imputation to reduce genotyping cost

Several sequencing platforms are available to obtain genotypic data. Parents of breeding populations may be genotyped at high density (HD) using an HD single‐nucleotide polymorphism (SNP) array or low‐pass sequencing (e.g., Li et al., 2021; Snelling et al., 2020; Song et al., 2013). However, given the relatively high cost of HD genotyping, it is uncommonly used to genotype the many thousands of strains typically created and tested in the preliminary stages of a plant breeding pipeline. Alternatives include medium‐density or LD SNP arrays (e.g., Song et al., 2020) and targeted genotyping‐by‐sequencing techniques such as molecular inversion probes (Wang et al., 2023). However, these LD genotyping methods may benefit from in silico genotyping methods that increase marker density by imputing from LD up to HD. For a continuously implemented GS pipeline, this process of genotyping thousands of strains needs to be completed within time and budgetary constraints to make timely selection decisions.

Several efficient methods are currently available for the imputation of genotypic information from LD to HD based on a reference panel of HD genotyped haplotypes (Gonen et al., 2018; Gorjanc et al., 2017; Swarts et al., 2014). A method implemented in AlphaPlantImpute (Gonen et al., 2018) uses a heuristic algorithm for phasing and imputing LD genotyped progeny populations using HD genotype information in parental strains. It is specifically designed to exploit features of a structured plant breeding program such as pedigree structure and patterns of recombination to improve imputation accuracy. We have implemented a wrapper around the Python implementation of AlphaPlantImpute (Gonen et al., 2018) using the *reticulate* R package (Ushey et al., 2024) to integrate it into the GS pipeline. AlphaPlantImpute can be implemented for pedigree‐based and population‐based imputation. When the pedigree of the strain to be imputed is known, and the parental lines are present in the HD reference panel, AlphaPlantImpute uses that information to perform phasing and imputation. However, for the strains whose pedigrees are not known, it is possible to perform population‐based imputation, requiring significantly more computational resources to phase and impute. Based on our simulation studies (results not shown), we recommend AlphaPlantImpute for imputing from LD to HD and LD‐KNNi for imputing scattered missing scores.

### 2.4 Phenotypic data input

**FIGURE 3:**
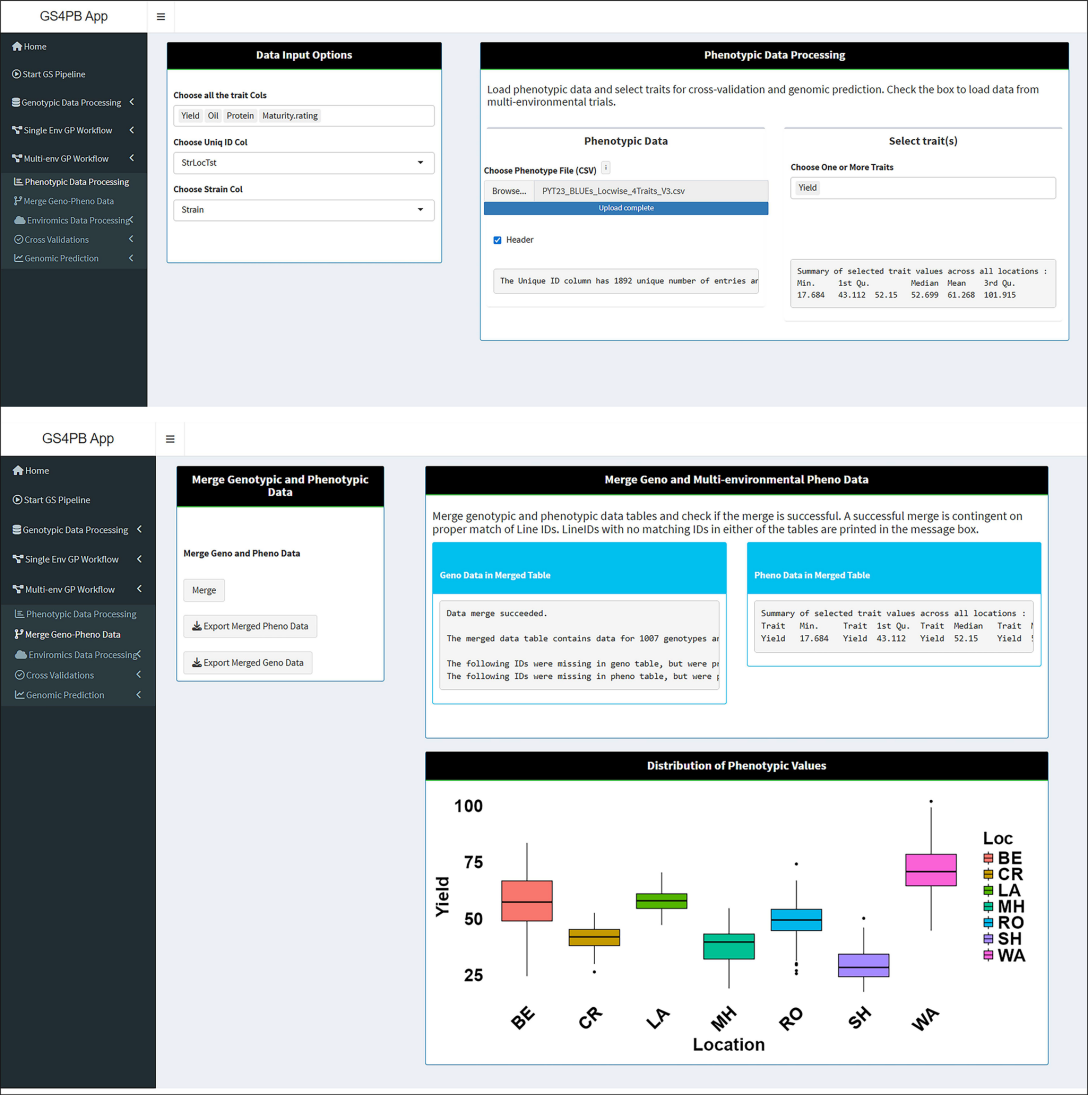
Screenshots of the GS4PB (Genomic Selection For Plant Breeding) app pages displaying the loading of phenotypic data (top panel) and merging of genotypic and phenotypic data (bottom panel). Phenotypic data are loaded in comma‐separated values (CSV) format (top panel). Once loaded, all the trait columns are selected in the sidebar panel, and the columns corresponding to the unique ID and strain ID are set. After setting these inputs, the trait(s) for cross validation and genomic prediction are selected. The bottom panel displays the step where the genotypic and phenotypic data tables are merged. Basic statistics for the genotypic and phenotypic data tables from the merged set are displayed. In the same panel, a box plot for the distribution of the selected trait values across environments is displayed. BE, Becker; CR, Crookston; GP, genomic prediction; LA, Lamberton; MH, Moorhead; RO, Rosemount; SH, Shelly; WA, Waseca.

In the current version of the pipeline, the application implements a two‐stage GS wherein best linear unbiased estimates (BLUEs) of strain effects are calculated using analysis tools outside of GS4PB such as ASReml‐R (Butler et al., 2023). We recommend BLUEs for genomic prediction model training to avoid the issue of “double shrinkage” (Holland & Piepho, 2024). If phenotypic data are from a multi‐environment trial (MET), BLUEs would be calculated for each of the lines in each of the environments using a model outside GS4PB, accounting for effects pertaining to the field trial design. Once the file containing the BLUEs of breeding line effects is uploaded in “.csv” format, the application displays the summary of trait values across all the environments or the distribution of trait values in each of the environments (Figure 3). Once trait values are selected, a summary of the trait values is printed.

### 2.5 Merging of genotype and phenotype data tables

In the next step, phenotypic and genotypic data are merged to form a data table, and lines with missing values for the selected trait for prediction are removed from the data table (Figure 3). This step ensures that the strain identifiers match in the uploaded genotypic and phenotypic data files.

### 2.6 “Enviromics” data processing

Integration of environmental data with genomic and high‐throughput phenotyping technology has been proposed as a way to improve prediction of G × E interaction effects (Crossa et al., 2024, 2022). Several studies have explored ways to integrate environmental covariates into genomic prediction models for improved accuracy (Canella Vieira et al., 2022; Costa‐Neto et al., 2021; Granato et al., 2018; Jarquin et al., 2014). However, several challenges remain, limiting the improvements in accuracy from such integration of environmental covariates (Crossa et al., 2024, 2022).

One of the challenges is obtaining high‐quality weather and soil data from all the locations in a multi‐environmental trial. While it is always a good idea to collect weather data and other environmental covariates on‐site, it is quite expensive for most programs. Weather stations are also prone to errors because of unforeseen circumstances or user error (Crossa et al., 2022; Monteiro et al., 2018). Public databases that collect satellite‐based weather data can serve as alternatives to obtain such data. We incorporated the *EnVRtype* package (Costa‐Neto et al., 2021), an R package that implements a pipeline to collect environmental data from public databases like NASA POWER (National Aeronautics and Space Administration‐Prediction of Worldwide Energy Resources) using the *nasapower* R package (Sparks, 2018) and others to collect weather data given the location coordinates and time window.

**FIGURE 4:**
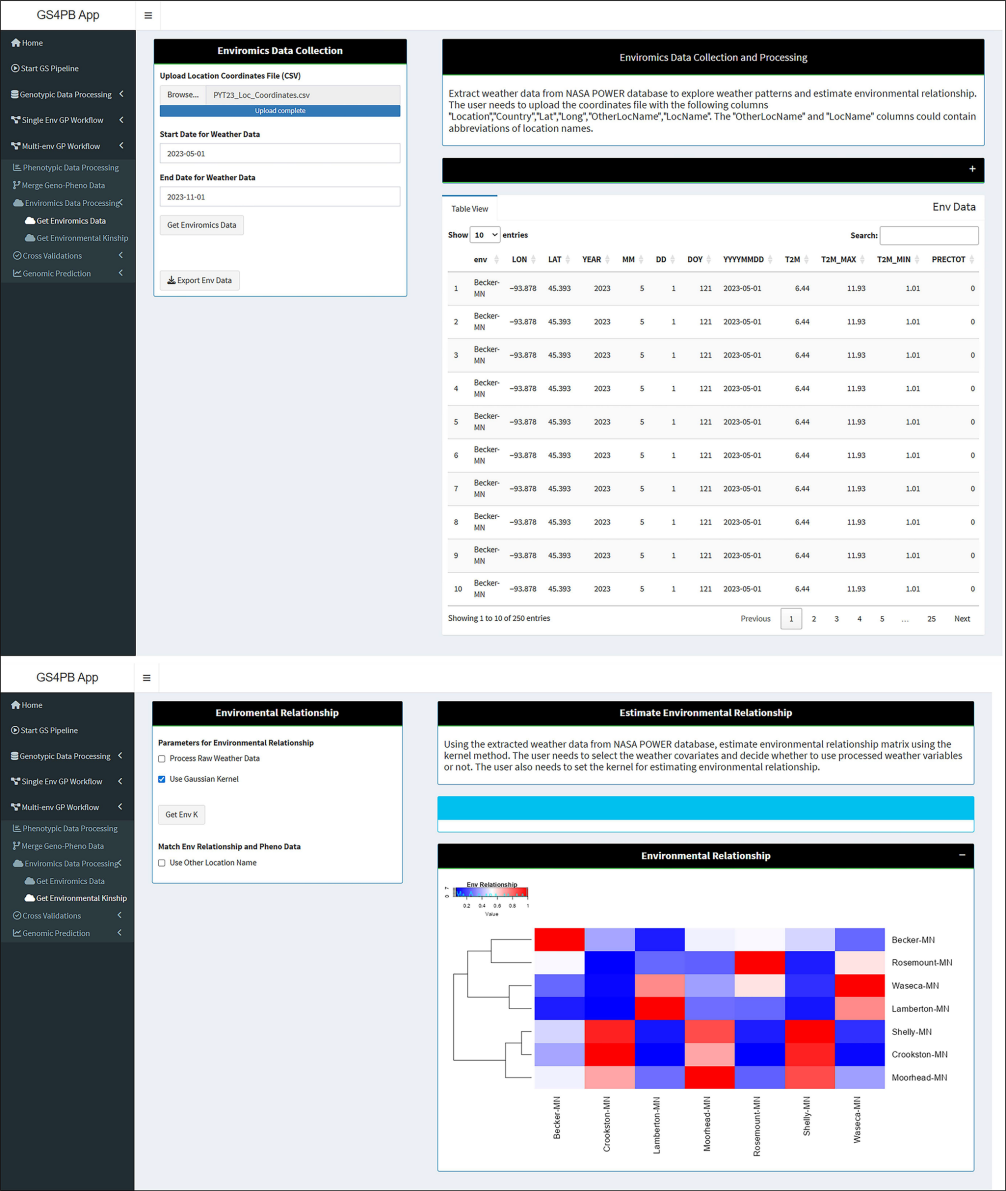
Screenshots of GS4PB (Genomic Selection For Plant Breeding) app enviromics data (top panel) and environmental relationship panels (bottom panel). In the enviromics panel, a location coordinate file in comma‐separated values (CSV) format is uploaded, and the start and end dates are set to define the time window as input. Once the environmental data are retrieved from the database, a table is displayed showing all the environmental variables that are extracted. In the bottom panel, the user can check the Gaussian kernel if required. Upon pressing the “GetEnv K” button, an environmental relationship kernel is estimated, and a heatmap depicting the environmental relationships is displayed. NASA POWER, National Aeronautics and Space Administration‐Prediction of Worldwide Energy Resources. GP, genomic prediction.

The collected weather data can be processed and used to estimate environmental relationship kernels using a linear or Gaussian kernel method to account for nonlinear effects (Costa‐Neto et al., 2021; Granato et al., 2018) (Figure 4). One of the challenges in this step is to identify informative environmental covariates relevant to the growth and development of a given crop to include in the environmental relationship estimation. Including naïve environmental covariates into genomic prediction models has been shown to be ineffective at improving prediction accuracies (Gevartosky et al., 2023; Jarquin et al., 2021). We plan to address this issue using a partial‐least squares approach (O. A. Montesinos‐López et al., 2022; Rebollo et al., 2023) and ML/AI techniques (O. A. Montesinos‐López et al., 2024; Shook et al., 2021) to identify the most relevant variables and critical time windows to refine the estimation of environmental kinship to be used in the linear mixed models. This feature will be included in future versions of GS4PB.

### 2.7 Training population optimization

Given the input target population of strains, which by default is the input training population, it is possible to select a subset of this as a training population for CV and genomic prediction. The user may set the training population size and select an optimization criterion such as “PEVMean” (mean of the prediction error variance), “PEVMax” (max of the prediction error variance), or “CDMean” (coefficient of determination mean) (Akdemir, in press; Akdemir et al., 2021; Mrode, 2014; Rincent et al., 2012). A training population for genomic prediction is selected by an objective function that optimizes the selected criteria using a genetic algorithm. Optimization can be performed against a target population or without any specification of the target population. The optimization method uses the “GenAlgForSubsetSelection” function implemented in the *STPGA* (Selection of Training Population Using Genetic Algorithms) R package (Akdemir, in press). The user also has the option to set parameters for the genetic algorithm if the default settings are not desired. By default, a CV is performed for the genomic prediction models using the optimized training population and a randomly selected training population of a specified size. Based on whether an optimal training population shows improved prediction accuracy, it is possible to set this optimal training population, or a randomly selected subpopulation as the training population for model training. The optional training population optimization step is currently not available for the multi‐trait and multi‐environment scenarios.

### 2.8 Genomic prediction and assessing prediction accuracy through CV

For single‐trait prediction, the ridge regression best linear unbiased prediction method from the *rrBLUP* package (Endelman, 2011), and “emRR” (expectation maximization—ridge regression), “emBayesB” (expectation maximization—Bayes B), and “emBayesLASSO” (expectation maximization least absolute selection and shrinkage operator) methods from the *bWGR* (Bayesian whole‐genome regression) package (Xavier et al., 2020) are implemented. In the current version, it is possible to perform *k*‐fold CV for any of these single‐trait models and select the best‐performing model for single‐trait prediction. For single‐trait genomic prediction, there are additional options to fit covariates as fixed effects and estimate the prediction error variance using the “kin.blup” function in *rrBLUP* (Endelman, 2011).

Multi‐trait prediction, which exploits genetic correlations between traits in addition to the genomic relationships among lines, uses multiple trait values simultaneously. This has the potential to improve accuracy relative to single‐trait prediction, especially for predicting traits with low heritability that are correlated with high heritability traits (Jia & Jannink, 2012; Lado et al., 2018; A. Montesinos‐López et al., 2016; Vitale et al., 2024). For multi‐trait prediction, Bayesian ridge regression, reproducing kernel Hilbert space, and spike‐slab regression methods implemented in the *BGLR* (Bayesian generalized linear regression) package (Perez‐Rodriguez & de Los Campos, 2022) are used for prediction. The application has a feature that allows the user to perform *k*‐fold CV in CV1 and CV2 schemes for these models and select the best‐performing model for making multi‐trait predictions. In the CV1 scheme, all traits in the target population lines are considered unknown and are predicted using models trained with information for both the focal and secondary traits exclusively from the training set. However, in the CV2 scheme, secondary traits from the target population are included in the training population to train the genomic prediction models. This significantly improves the ability to predict the values of unknown focal traits that are correlated with the secondary traits, whose values are known in both the training and target populations (Burgueño et al., 2012; Lado et al., 2018; Runcie & Cheng, 2019).

**FIGURE 5:**
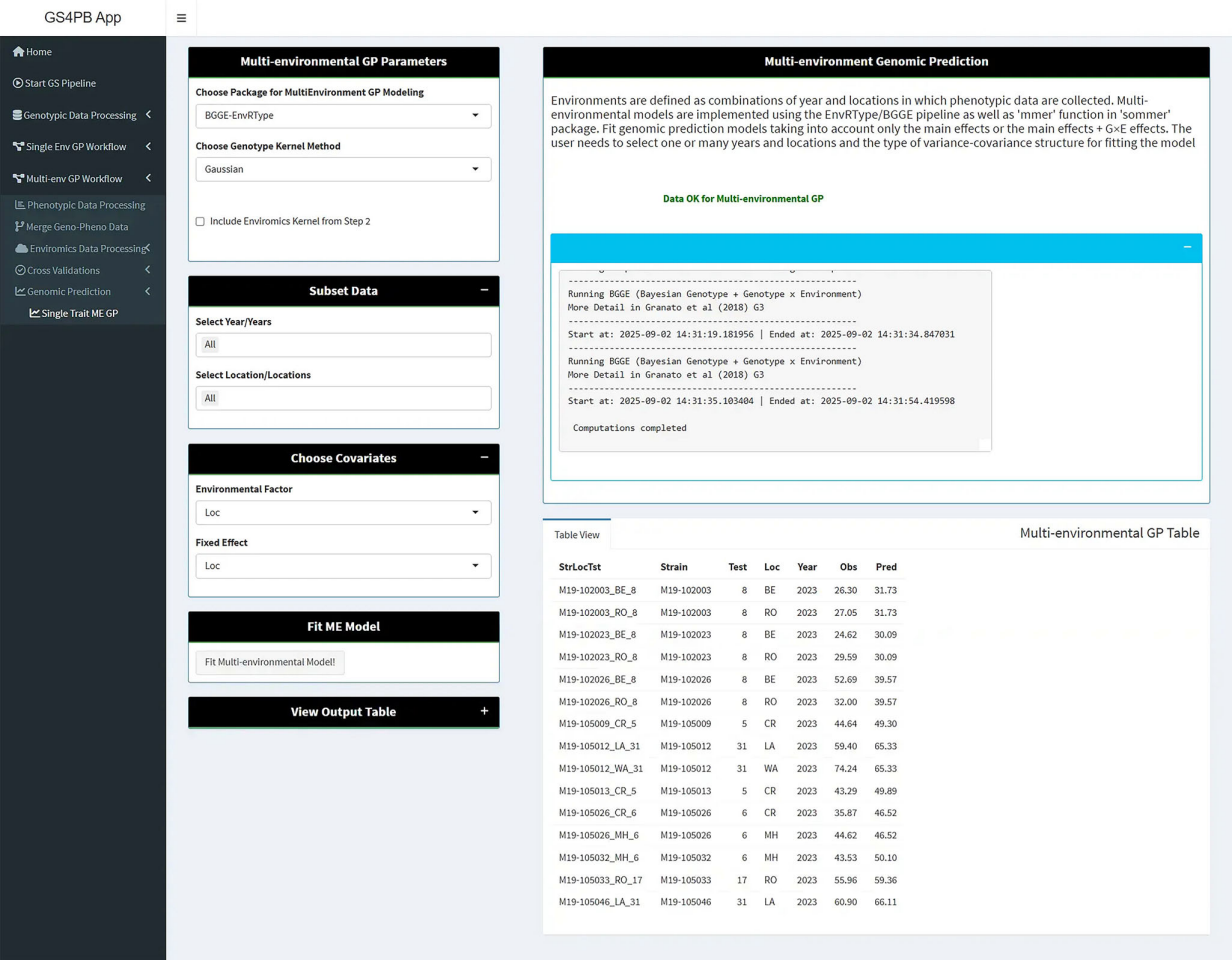
Screenshots of the GS4PB (Genomic Selection For Plant Breeding) page for multi‐environmental genomic prediction (ME‐GP; bottom panel). In the ME‐GP panel, the user can select years, locations, and set covariates such as the data column corresponding to the environmental factor and fixed effect. In the current implementation, only the BGGE‐EnvRtype (where BGGE stands for Bayesian genotype and genotype‐by‐environment) package is selected. The user has the option to select a linear or Gaussian kernel method for estimating genomic relationships between strains. Once the GP model is fit, a table containing the observed and predicted values for the selected model is displayed, and the table can also be downloaded from the app. GP, genomic prediction.

For multi‐environmental data, genomic prediction models that consider G × E interaction effects show better accuracy, especially for predicting the performance of lines that differ with environments (Costa‐Neto et al., 2021; Crossa et al., 2022; Granato et al., 2018; Jarquin et al., 2014). For multi‐environment data, the methods implemented in the *BGGE* (Bayesian genotype and genotype‐by‐environment) and *EnvRtype* (Costa‐Neto et al., 2021; Granato et al., 2018) packages are used in the application to model G × E interaction effects. In the current implementation of this method, three models are trained with or without an environmental relationship matrix (Figure 5): (i) the “main effects” model, which includes only the main effects of strains and environments (Y = G + E); (ii) the MDs (main effects model with single deviations) model, which includes the main effects in the previous model and a term for G × E interaction that are estimated as random deviations with a common variance (Y = G + E + G × E); and (iii) the MDe (main effects model with environment‐specific deviations) model includes the terms in the main effect model and random deviations for the G × E term with environment‐specific variances/covariances (Y = G + E + G × E*_i_*).

For multi‐environment genomic prediction models, the following cross‐validation schemes are currently implemented: CV2, where tested strains in tested environments are predicted; CV1, where untested strains in tested environments are predicted; CV0, where tested strains in untested environments are predicted; CV00, where untested strains in untested environments are predicted; and CV LOFO, where one factor chosen by the user is left out (Granato et al., 2018; Jarquin et al., 2014). Factors are flexible and could include any covariate in which the user wants to divide the data for validating models, with the more commonly used ones in this context being environment or trial. For example, if strains are organized into different trials, then a trial could be defined as a factor, and the algorithm would leave a complete trial out, build a model using all other trials in the dataset, and predict the trial left out.

## 3 SOFTWARE AND DEPLOYMENT

The application is written in R using the R *shiny* package (Chang et al., 2025). The application is roughly split into modules to implement each of the steps in the pipeline. A modular framework allows easy modifications, additions, and integration of new tools as required. The user interface is written using R packages such as *shinyjs* (Attali, 2021) and *shinydashboard* (Chang & Borges Ribeiro, 2021) that supplement the functions in R *shiny* to provide better ways of organizing elements of the user interface. The app is organized into an R package using the golem framework (Fay et al., 2024). This simplifies the distribution of the app, as any R package can be made available in CRAN and can also be installed and used like any other R package. For more advanced users, it also allows implementation of more complex pipelines with steps that require finer parameter tuning using the package instead of the user interface. A containerized version of the same application is available on Docker Hub (https://hub.docker.com/r/umnlorenzgroup/gs4pb). Source code can be found here: https://github.com/UMN‐Lorenz‐Group/GS4PB.git. Documentation for installing and running the application can be found here: https://github.com/UMN‐Lorenz‐Group/GS4PB/wiki.

Performance results for genotypic data of different sizes demonstrate the feasibility of using GS4PB on larger datasets (Table S1). An easy‐to‐use deployment that facilitates a smooth and efficient multiuser experience is another important factor in improving the rate of user adoption. Our current implementation is available on Docker Hub as a container image as well as a GitHub repository. The current recommended method for running the application is via the Docker container, as it helps users avoid installing (and reinstalling) all the packages in their computing environment. However, the performance of the application, when run from the container, depends on the user system and may not be ideal for intensive computation. So, we have also deployed the application on CyVerse DE–VICE (CyVerse Discovery Environment–Virtual Interactive Computing Environment) (Swetnam et al., 2024) (https://de.cyverse.org//apps/de/4a15ab6a‐a2b5‐11ef‐8547‐008cfa5ae621). On Cyverse, which is one of the largest open‐source research cyberinfrastructures, every registered user can request computing resources while running applications such as R Shiny applications. For developers, CyVerse also provides support for continuous development and integration on cloud platforms. However, the requirement of registration and login information might limit some users from using the application on CyVerse. Hosting the application on “PositConnect” is another option we would like to explore for such users. By deploying on multiple platforms, we hope to encourage user adoption of the application and lower the technical barrier to implementing GS.

## 4 USAGE EXAMPLE

### 4.1 Overview

We set out to evaluate the implementation of the GS pipeline described above using data from preliminary yield trials (PYTs) conducted in 2023 as part of the University of Minnesota Soybean Breeding Program. These early‐stage yield trials are used to discard a large fraction of breeding lines of relatively poor performance in their first year of testing and advance a smaller fraction to a second year of testing, typically called advanced yield trials (AYTs). Application of genomic prediction at this stage could save money in multiple ways. One such way may be to reduce phenotyping costs by phenotyping only a subset of the PYT‐stage breeding lines, genotyping all breeding lines with genome‐wide markers, training a genomic prediction model using data from the phenotyped subset, and finally using the genomic prediction model to predict the performance of all breeding lines. Breeding lines would be advanced to AYTs on the basis of their genomic prediction. There are many other options to using predictions at this stage to increase program efficiency, including using genomic prediction combined with sparse testing to reduce plot resources while possibly maintaining or improving selection accuracy (Jarquin et al., 2020; Persa et al., 2023).

### 4.2 Description of germplasm and field design

The germplasm used for this case study consisted of 932 F_4:6_ soybean lines entered into the 2023 PYTs. These breeding lines were derived from 105 unique families (biparental populations), with a mean of 9.8 lines from each family. As is typical for a diverse public program, the parents used to create the families were selected for a variety of traits and target markets, including high yield, resistance to soybean cyst nematode, seed protein, seed quality and size, high oleic fatty acid, and resistance to soybean aphid, among other traits. Relative maturity of the breeding lines varied from approximately 0.9 to 1.9.

Each of the 932 breeding lines was tested at two locations in one of three maturity zones in Minnesota (Figure S1) in 2023. Lines were placed in maturity zones based on their relative maturity estimated in the prior year at the progeny row stage. Early relative maturity lines were tested in the northern zone at trials sites nearby Crookston, MN (47.8° N), Shelly, MN (47.5° N), and Moorhead, MN (46.9° N). Intermediate relative maturity lines were tested in the central zone at trials sites nearby Becker, MN (45.4° N), and Rosemount, MN (44.7° N). Lines of later relative maturity were tested in the southern zone at trials sites nearby Waseca, MN (44.1° N), and Lamberton, MN (44.2° N). Within each location, the breeding lines were organized into trials that consisted of lines with similar target product profiles (e.g., tofu/soymilk). Each trial consisted of 32–48 entries, including three performance and maturity checks that were common across trials. Each trial was designed as a randomized complete block design with two replications. Plots consisted of two rows 3.66 m in length spaced 0.76 m apart.

Seed yield was measured as total seed weight per plot standardized to 13% moisture and converted to kilograms per hectare. The date of maturity of each plot was determined as the date on which 95% of the pods had reached their mature color. Because days to maturity is correlated with seed yield, all plot‐level seed yield data points were adjusted using maturity date as a covariate to account for variation in maturity date. The following linear model fitted the data from each location ‘l’ to obtain best linear unbiased estimates of breeding line effects:

(1) $$y_{ijk}=\mu +g_{i}+t_{j}+b_{k\left(j\right)}+\varepsilon_{ijk}$$

where μ is the intercept, $g_{i}$ is the effect of the *i* ^th^ breeding line, $t_{j}$ is the effect of the *j* ^th^ trial, $b_{k(j)}$ is the effect of the *k* ^th^ block nested within the *j* ^th^ trial, and $\varepsilon_{ijk}$ is the residual. All effects but for the residual were fit as fixed effects using ASReml‐R (Butler et al., 2023) and BLUEs of breeding line effects were output and later used in genomic prediction models.

### 4.3 Environmental kinship

Weather data in the form of average daily measurements for the time window from May 1, 2023, to November 30, 2023, for the 2023 PYT locations were extracted from the NASA POWER database using wrappers implemented in the *EnvRtype* package (Costa‐Neto et al., 2021; Sparks, 2018). The weather variables collected were “T2M,” “T2M_MAX,” “T2M_MIN,” “T2MDEW,” “ALLSKY_SFC_LW_DWN,” “ALLSKY_SFC_SW_DWN,” “ALLSKY_SFC_SW_DNI,” “ALLSKY_SFC_PAR_TOT,” “ALLSKY_SFC_PAR_TOT,” “ALLSKY_SFC_PAR_UVA,” “ALLSKY_SFC_PAR_UVB,” “PRECTOT,” “WS2M,” “RH2M,” and “EVPTRNS,” which correspond to measurements of temperature, radiation, precipitation, wind speed, relative humidity, and evapotranspiration (Figure S2; Table S2). Using these data, an environmental kinship matrix was estimated using a Gaussian kernel. The locations cluster together into the three zones for 2023 (Figure S3).

### 4.4 Genotyping, imputation, and quality verification

Forty‐nine parental lines of the breeding lines entered into the 2023 PYTs were genotyped using the Gencove low‐pass sequencing platform (Li et al., 2021; Snelling et al., 2020), which generated approximately 27.7 million variants at an average coverage of 0.4–0.5x, including approximately 25 million SNPs. A subset of 32.8K variants corresponding to the SOYSNP50K (Song et al., 2013) assay was extracted for use as an HD genotyping parental panel. The 2023 PYT breeding lines were assayed using the Agriplex Soy 1K SNP assay (https://www.agriplexgenomics.com/soybean‐community‐panel). This 1K SNP set, after filtering, was imputed up to the 32.8K set using the AlphaPlantImpute (Gonen et al., 2018) population‐based imputation method. The imputed 32.8K SNP set was used to estimate the genomic kinship matrix of PYT lines.

A genotypic data quality check is highly recommended when combining genotyping data from different platforms/assays. For example, in this case study, the Agriplex Soy 1K SNP calls were aligned against the Glyma.Wm82.a1.v1 assembly, whereas the Gencove genotypic data were called against the Glyma.Wm82.a4.v1 assembly. Such differences in assembly versions necessitate a liftover process to map the coordinates to the more recent assembly and check reference calls against that specific reference assembly using a tool such as the “fixref/checkref” utility available as part of BCFtools (Danecek et al., 2021). Liftover was performed using the Mummer/CrossMap pipeline (Marçais et al., 2018; Zhao et al., 2014). Considering all these transformations to the input genotypic data, it is advisable to check the integrity of genotypic data before and after processing.

The integrity of genotypic data generated using the Agriplex Soy 1K SNP assay and the genotypic data imputed up to the HD SNP set using AlphaPlantImpute was assessed using principal component (PC) analysis to help ensure no changes in estimated genomic relationships among lines were introduced. For both SNP sets, the relative spatial positions of parents and progenies were similar on the two‐dimensional PC plot (Figure S4). The correlations of the loadings on PC1 and PC2 between the LD SNP set and imputed HD SNP set were 0.84 and 0.83, respectively. This result suggests that the imputation and translation between different genome assemblies preserved the relative genetic distances between breeding lines.

### 4.5 Leave‐one‐test‐out CV

We performed a leave‐one‐trial‐out (LOTO) CV on the yield data from the PYTs for an initial assessment of predictive ability (PA) using the analysis workflow of the GS4PB R Shiny application. “Trials” were comprised of a unique set of breeding lines evaluated for yield and days to maturity at two locations. In this LOTO CV, when a trial was removed from the training dataset, a unique set of genotypes is assumed to be unknown and are predicted using models trained on data from genotypes of the remaining trials. This CV scheme allows one to assess the ability to predict novel genotypes in tested environments.

We compared a set of models available within the GS4PB app, including a main effects model (Y = G + E), main effects plus a G × E interaction effect (Y = G + E + G × E), and a main effects plus G × E interaction effects model assuming heterogenous genotypic variances across environments (Y = G + E + G × E*_i_*). We also included a covariate (W) in the form of an environmental kinship matrix estimated from the weather data in these three models to evaluate the enviromics‐enriched versions of these models. For this, we used custom scripts in GS4PB to implement methods described in the *BGGE* and *EnvRtype* packages (Costa‐Neto et al., 2021; Granato et al., 2018) to fit a kernel‐based Bayesian G‐BLUP model with and without enviromics covariates. Both linear and Gaussian kernels were compared for the genomic kernel, whereas a common Gaussian kernel was used for capturing environmental relationships. A detailed description of each model is provided in Table S3.

Predictive abilities of the various model options available in GS4PB were highly similar in most cases (Table 1). Models assuming homogenous genetic variances across environments ranged in PA from 0.469 (G + E + W + G × W, Gaussian genomic kernel) to 0.435 (G + E + G × E + W + G × W, linear genomic kernel), which was less than the standard error of each estimated mean PA. The inclusion of environmental covariates did not improve PA for this dataset, indicating more research is needed on identifying and weighting the most informative environmental covariates for genomic prediction. Optimizing the choice and weighting of the enviromic covariates was outside the scope of this study. Modeling heterogenous genetic variances across environments actually lowered PA, most likely because of the limited data within each environment to accurately estimate each unique genetic variance component, introducing error into the model and thus lowering PA. Overall, the results of the different models illustrate the ability of each model to produce fairly accurate predictions. Future research will help refine these models, especially the G × E models that incorporate the enviromic data.

**TABLE 1:** Predictive ability of alternative genomic prediction models incorporating genotype (G), environment (E), enviromics (W), and their interaction terms. Each model specifies random effects parameterized with appropriate covariance structures based on genomic, environmental, or enviromic kernels. Genomic kinship was estimated using both Gaussian and linear kernel methods, whereas environmental kinship was estimated from weather covariates using only a Gaussian kernel. For each model, kernel combination, the mean predictive ability, standard error of the mean, and observed range (minimum [Min] and maximum [Max]) are displayed.

Model	Genomic kernel estimation method	Predictive ability (PA)	Standard error of PA estimate	Min PA across trials	Max PA across trials
G + E	Gaussian	0.439	0.114	0.165	0.760
	Linear	0.451	0.111	0.195	0.783
G + E + G × E	Gaussian	0.464	0.073	0.302	0.713
	Linear	0.461	0.076	0.296	0.734
G + E + G × E*_i_*	Gaussian	0.360	0.105	0.053	0.703
	Linear	0.402	0.073	0.245	0.662
G + E + W	Gaussian	0.439	0.114	0.168	0.762
	Linear	0.453	0.110	0.198	0.785
G + E + G × E + W	Gaussian	0.461	0.070	0.296	0.691
	Linear	0.459	0.075	0.294	0.727
G + E + G × E*_i_* + W	Gaussian	0.332	0.054	0.159	0.465
	Linear	0.340	0.091	0.044	0.588
G + E + W + G × W	Gaussian	0.469	0.077	0.310	0.744
	Linear	0.458	0.078	0.309	0.757
G + E + G × E + W + G × W	Gaussian	0.457	0.074	0.300	0.722
	Linear	0.435	0.077	0.300	0.729
G + E + G × E*_i_* + W + G × W*_i_*	Gaussian	0.328	0.111	−0.048	0.624
	Linear	0.382	0.084	0.240	0.683

*Note*: Model formulae indicate the terms that are included in kernel‐based genome‐wide regression models. Environmental factor groupings (E) are modeled as fixed effects in all models. For all of the above models, the number of iterations, burn‐in, and thin parameters in the Gibbs sampler were set to 10,000, 2000, and 5, respectively, and other parameters were set at the default value in the BGGE (Bayesian genotype and genotype‐by‐environment) package.

### 4.6 Comparing genomic and phenotypic selection using divergent selection

The efficacy of the GS analysis pipeline implemented in GS4PB was further evaluated using replicated, multilocation field trials in 2024 in the central and southern Minnesota zones. Briefly, both genomic predictions for yield output from the GS4PB pipeline and BLUEs of breeding line effects for yield from field evaluations in 2023 were used to select 20 low‐yielding breeding lines and 20 high‐yielding breeding lines. Both genomic predictions and phenotypic BLUEs of yield were adjusted for maturity using maturity date as a covariate as described above. The breeding lines were grouped into their corresponding maturity zones and sorted based on the value of yield adjusted for maturity and ranked within zones. The top 20 ranked and the lowest ranked 20 breeding lines for the central and southern zones from both genomic prediction and phenotypic BLUEs were selected, creating the following selection treatment categories: “phenotypic selection high” (PS‐High), “phenotypic selection low” (PS‐Low), “genomic selection high” (GS‐High), and “genomic selection low” (GS‐Low). There were seven breeding lines common to the GS‐High and PS‐High selection treatments (three in the central zone and four in the southern zone), and 12 breeding lines common to the GS‐Low and PS‐Low selection treatments (three in the central zone and nine in the southern zone). Selected breeding lines, along with three performance checks, were grown in three Minnesota locations in two replicates in each of the central and southern zones in 2024. The southern zone locations were Lamberton, Waseca, and Lake Wilson. The central zone locations were Morris, Becker, and Rosemount. A linear model including location, breeding line, breeding line × location interaction, and replication nested within location was fit to the data from each zone separately. All effects, except for the residual, were treated as fixed effects. BLUEs of breeding line effects for yield were obtained from the model and used to compare the different selection treatments. We chose to include a low‐yield selection treatment because the culling of poor‐performing breeding lines before expensive field testing is a common application of GS (Wartha & Lorenz, 2021).

**FIGURE 6:**
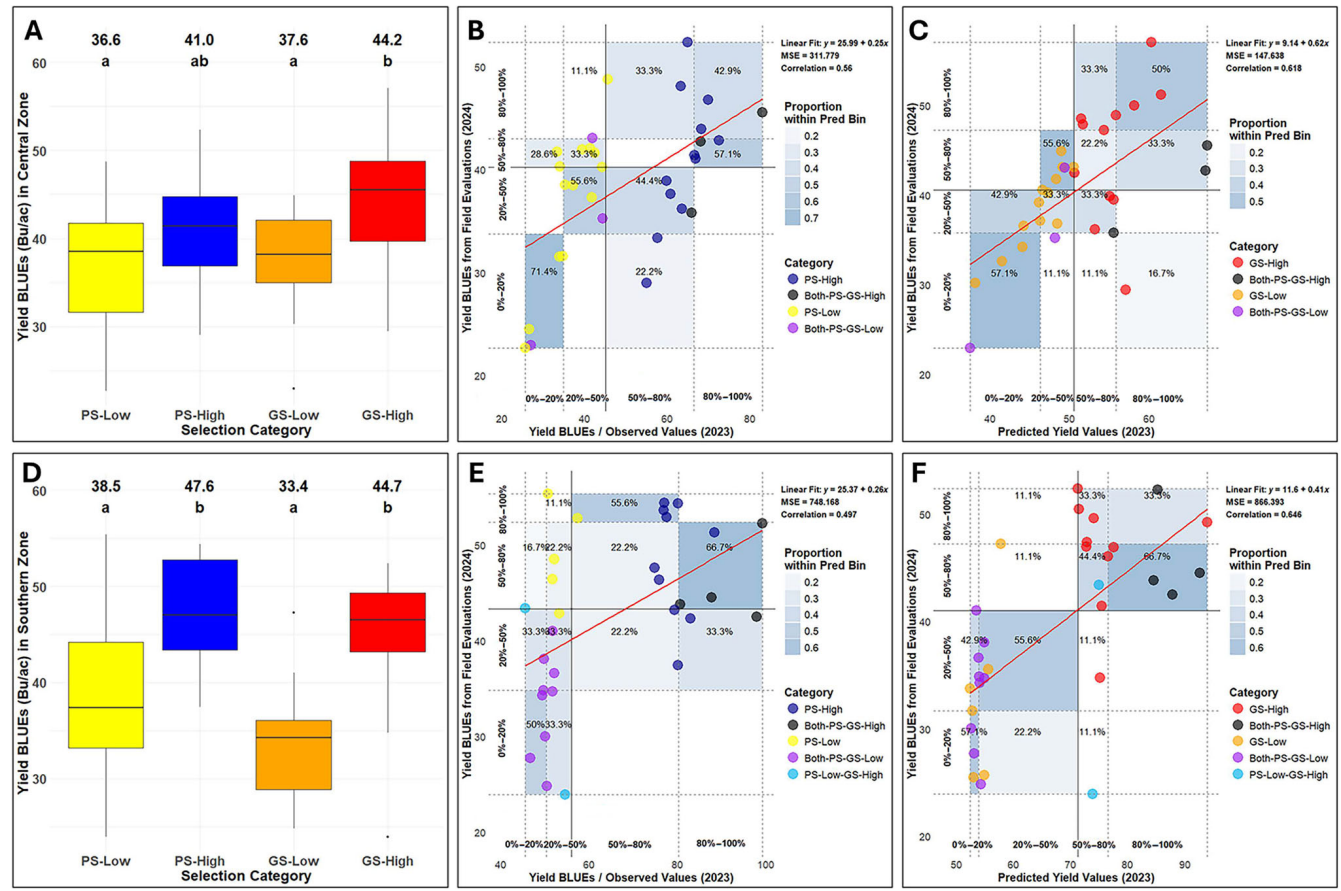
Results from a validation study comparing genomic selection using GS4PB (Genomic Selection For Plant Breeding) and phenotypic selection. Panels (A) and (D) display box plots comparing the different selection treatments in the central and southern Minnesota maturity zones, respectively: phenotypic selection for low yield (PS‐Low), phenotypic selection for high yield (PS‐High), genomic selection for low yield (GS‐Low), and genomic selection for high yield (GS‐High). The selection treatments were applied in 2023 and compared against one another using best linear unbiased estimates (BLUEs) of breeding lines effects for yield measured in a 2024 validation trial. Numbers at the top of the plots are the actual average yield values in bushels per acre (Bu/ac), and the letters underneath indicate statistical differences among the treatments, with treatment means not sharing a letter indicating statistically significant differences (*p* < 0.05) using Tukey's honestly significant difference. Panels (B) (central zone) and (E) (southern zone) compare the breeding line BLUEs based on 2023 preliminary yield trial data (*x*‐axis) to the 2024 validation trial yield BLUEs of breeding lines effects (*y*‐axis). Points are colored by the selection treatment applied based on the 2023 data. “Both‐PS‐GS‐High” indicates the breeding line was in the PS‐High treatment and GS‐High treatment, and likewise for “Both‐PS‐GS‐Low.” The “PS‐Low‐GS‐High” label indicates breeding lines in the PS‐Low treatment and GS‐High treatment (*N* = 2). Panels (C) (central zone) and (F) (southern zone) are similar to Panels (B) and (E) except that the *x*‐axis plots the genomic prediction value based on 2023 data. The *y*‐axis is the same as before. The selection categories are the same except that genomic predictions were used rather than phenotypic BLUEs. The plot also includes 20, 50, and 80 percentile grids along both the *x*‐ and *y*‐axes to visualize the percentage of lines that are present in each of these bins. Percentage values are reported relative to the predicted values bin on the *x*‐axis. For example, a value of 50% in the bin (80%–100% bin) on both *x*‐ and *y*‐axes in Panel (C) denotes that 50% of the predicted values in that bin are also observed to be in the same percentile bin in field evaluations. Since the proportions are relative to the predicted bins, the sum of all percent values along the *y*‐axis for each of the bins in the *x*‐axis will be 100%. None of the top performing lines are predicted to be in the worst performing bins in both the central and southern zones (note the absence of any point in the 0%–20% bin of predicted values in the 80%–100% as well as the 50%–80% bins of observed values in both the central and southern zones (Panels C and F). However, ∼43% and 50% of the lines in the central and southern zones, respectively, that are predicted to be in the 80‐100 percentile are also observed in that bin (Panels C and F). MSE, mean squared error.

Overall, genomic prediction implemented with GS4PB performed at least as well as PS in terms of identifying the high‐ and low‐yielding breeding lines for the advancement to the next stage of testing (Figure 6A,D). The GS‐High and PS‐High selection treatments were not significantly different from one another (*p* > 0.05), as was the case for the GS‐Low and PS‐Low selection treatments. The difference between GS‐High and GS‐Low was numerically greater than the difference between PS‐High and PS‐Low in both zones. Additionally, GS‐High and GS‐Low were significantly different from one another in the central zone (*P* < 0.05), whereas we did not observe a significant difference between PS‐High and PS‐Low.

A more detailed look at scatter plots comparing PSs and GSs to 2024 yield values further demonstrates how well GS implemented in GS4PB worked relative to PS (Figure 6B,C,E,F). Neither PS nor GS was perfect in identifying high‐ and low‐yielding lines in the 2024 validation trials, but GS tended to be superior. For example, 13 total breeding lines phenotypically selected for low yield in 2023 were in the top 50% for 2024 yield, but there were only eight such breeding lines advanced by GS. The correlation between 2023 and 2024 yield was better for the genomic predictions than phenotypic BLUEs, being 0.56 (central zone) and 0.50 (southern zone) for phenotypic BLUEs versus 0.62 (central zone) and 0.65 (southern zone) for the genomic predictions. Overall, this validation study showed that genomic prediction implemented via GS4PB worked at least as well as PS for advancing breeding lines to the next stage of yield testing in a soybean breeding program.

## 5 CONCLUSION

To our knowledge, the application we have developed and described above is the most comprehensive application implementing a genomic prediction and selection analysis workflow that is publicly available. All steps are implemented using various R packages that have been tested extensively for specific purposes. While there are a few apps that implement genomic prediction modeling such as solGS (Tecle et al., 2014), iPat (Chen & Zhang, 2018), and ChiDO (González et al., 2024) each with its unique strengths, these applications do not implement a comprehensive GS workflow.

In the future, we plan to integrate additional features including prediction of superior progeny means of all possible breeding crosses (Akdemir & Sanchez, 2016; Mohammadi et al., 2015; Wartha & Lorenz, 2024) and pedigree checks for quality control. We also plan to integrate ML/AI techniques for feature engineering of environmental covariates for improved prediction of G × E interaction effects. In addition, we plan to include features for “drag and drop” steps to design a pipeline and perform single‐click implementation to automate some of the steps. By including all these features, we hope to provide an easy‐to‐use application that comprehensively implements GS for routine use by breeders.

## AUTHOR CONTRIBUTIONS

**Vishnu Ramasubramanian**: Conceptualization; formal analysis; investigation; methodology; software; writing—original draft; writing—review and editing. **Cleiton A. Wartha**: Investigation; methodology; writing—review and editing. **Lovepreet Singh**: Investigation; methodology; writing—review and editing. **Paolo Vitale**: Investigation; methodology; writing—review and editing. **Sushan Ru**: Methodology; writing—review and editing. **Siddhi J. Bhusal**: Data curation; methodology. **Aaron J. Lorenz**: Conceptualization; funding acquisition; investigation; methodology; project administration; supervision; writing—review and editing.

## CONFLICT OF INTEREST STATEMENT

The authors declare no conflicts of interest.

## Supporting information



Supplementary Material

Supplementary Material

Supplementary Material

Supplementary Material

## Data Availability

All source code and example datasets can be found at https://github.com/UMN‐Lorenz‐Group/GS4PB. A containerized version of the same application is available on Docker hub: https://hub.docker.com/r/umnlorenzgroup/gs4pb. Documentation for installing and running the application can be found here: https://github.com/UMN‐Lorenz‐Group/GS4PB/wiki.
