# Supplementary material for: GS4PB: An R Shiny application to facilitate a genomic selection pipeline for plant breeding
Source: Plant Genome. 2025 Dec 11;18(4):e70150. doi: 10.1002/tpg2.70150 (PMC12698896; doi:10.1002/tpg2.70150)
Supplement: Supplementary file 1 [file TPG2-18-e70150-s001.docx]

**Supplementary Table 2.** Description of weather covariables used to estimate an “enviromics kinship matrix” for the 2023 PYT locations. The information is sourced from ‘nasapower’ and ‘get_weather’ function documentation (Sparks 2018; Costa-Neto et al., 2021).

| **Weather Variable Acronym** | **Description** | **Units** |
| --- | --- | --- |
| T2M | Temperature at 2 Meters | C |
| T2M_MAX | Maximum Temperature at 2 Meters | C |
| T2M_MIN | Minimum Temperature at 2 Meters | C |
| PRECTOT | Precipitation | mm |
| WS2M | Wind Speed at 2 Meters | m/s |
| RH2M | Relative Humidity at 2 Meters | percentage |
| T2MDEW | Dew/Frost Point at 2 Meters | C |
| ALLSKY_SFC_LW_DWN | Downward Thermal Infrared (Longwave) Radiative Flux |  |
| ALLSKY_SFC_SW_DWN | All Sky Insolation Incident on a Horizontal Surface |  |
| ALLSKY_SFC_SW_DNI | All Sky Surface Shortwave Downward Direct Normal Irradiance |  |
| ALLSKY_SFC_UVA | All Sky Surface Ultraviolet A (315nm-400nm) Irradiance |  |
| ALLSKY_SFC_UVB | All Sky Surface Ultraviolet B (280nm-315nm) Irradiance |  |
| ALLSKY_SFC_PAR_TOT | All Sky Surface Photosynthetically Active Radiation (PAR) Total |  |
| FROST_DAYS | If it was a frost day (temperature less than 0C or 32F) |  |
