# Supplementary material for: GS4PB: An R Shiny application to facilitate a genomic selection pipeline for plant breeding
Source: Plant Genome. 2025 Dec 11;18(4):e70150. doi: 10.1002/tpg2.70150 (PMC12698896; doi:10.1002/tpg2.70150)
Supplement: Supplementary file 2 [file TPG2-18-e70150-s002.docx]

Supplementary Table 3: Genomic prediction models incorporating genotype (G), environment (E), enviromics (W), and interaction terms. The random terms and variance-covariance kernels for the specific main and interaction effects are described in the model description column.

| Model | Model description †† |
| --- | --- |
| G+E | Main effects model. Genotype effects are modelled as random terms, distributed as ~ N (0, Z_g_K_g_Z_g_’) |
|  |  |
| G+E+GxE | Main effects and single homogeneous variance for GxE deviations (MDs). Genotype and genotype x environment effects are modelled as random terms, with genotype effects distributed as ~N (0, Z_g_K_g_Z_g_’) and genotype x environment effects distributed as ~ N (0, Z_g_K_g_Z_g_’ o Z_e_IZ_e_’) |
|  |  |
| G+E+GxE_i_ | Main effects and environment specific heterogeneous variance (MDe) for GxE deviations. Genotype effects and genotype x environment effects are modelled as random terms, with genotype effects distributed as ~N (0, Z_g_K_g_Z_g_’) and genotype x environment effects distributed as ~N (0, Z_g_K_g_Z_g_’ o Z_ei_IZ_ei_’) |
|  |  |
| G+E+W | Enviromics enriched MM, main effects model with both genotype and enviromics effect. Genotype and enviromics effects are modelled as random terms with genotype effects distributed as ~N (0, Z_g_K_g_Z_g_’) and enviromics effects distributed as ~N (0, Z_e_K_e_Z_e_’) |
|  |  |
| G+E+GxE+W | Enviromics enriched MDs model. Genotype, genotype x environment and enviromics effects are modelled as random terms, with genotype effects distributed as ~ N (0, Z_g_K_g_Z_g_’), genotype x environment effects distributed as ~N (0, Z_g_K_g_Z_g_’ o Z_e_IZ_e_’) and enviromics effects distributed as ~ N (0, Z_e_K_e_Z_e_’) |
|  |  |
| G+E+GxE_i_+W | Enviromics enriched MDe model. Genotype, genotype x environment and enviromics effects are modelled as random terms, with genotype effects distributed as ~N (0, Z_g_K_g_Z_g_’), genotype x environment effects distributed as ~N (0, Z_g_K_g_Z_g_’ o Z_ei_IZ_ei_’) and enviromics effects distributed as ~ N (0, Z_e_K_e_Z_e_’) |
|  |  |
| G+E+W+GxW | Reaction norm model with main effects. Genotype, enviromics, and genotype x enviromics effects are modelled as random terms, with genotype effects distributed as ~N (0, Z_g_K_g_Z_g_’), enviromics effects distributed as ~ N (0, Z_e_K_e_Z_e_’) and genotype x enviromics effects distributed as ~N (0, Z_g_K_g_Z_g_’ o Z_e_K_e_Z_e_’) |
|  |  |
| G+E+GxE+W+GxW | Reaction norm model with MDs. Genotype, genotype x environment, enviromics and genotype x enviromics effects are modelled as random terms, with genotype effects distributed as ~N (0, Z_g_K_g_Z_g_’), genotype x environment effects distributed as ~N (0, Z_g_K_g_Z_g_’ o Z_e_IZ_e_’), enviromics effects distributed as ~ N (0, Z_e_K_e_Z_e_’) and genotype x enviromics effects distributed as ~N (0, Z_g_K_g_Z_g_’ o Z_e_K_e_Z_e_’) |
|  |  |
| G+E+GxE_i_+W+GxW_i_ | Reaction norm model with MDe. Genotype, genotype x environment, enviromics and genotype x enviromics effects are modelled as random terms, with genotype effects distributed as ~N (0, Z_g_K_g_Z_g_’) and genotype x environment effects distributed as ~N (0, Z_g_K_g_Z_g_’ o Z_ei_IZ_ei_’), enviromics effects distributed as~ N (0, Z_e_K_e_Z_e_’) and genotype x enviromics effects distributed as ~N (0, Z_g_K_g_Z_g_’ o Z_ei_K_e_Z_ei_’) |

†† Z_g_ refers to the genotype indicator matrix in observation space with dimensions n*p, where ‘n’ is the number of observations and ‘p’ is the number of genotypes. Z_e_ refers to the environment indicator matrix of dimensions n*l, where ‘n’ is the number of observations and ‘l’ is the number of environments. K_g_ denotes a genomic relationship matrix estimated using a linear or Gaussian kernel function. K_e_ refers to an “environmental kinship” matrix estimated using enviromic weather covariates with a Gaussian function. ‘I’ refers to an identity matrix of dimensions n*n. ‘o’ in (Z_g_K_g_Z_g_’ o Z_e_IZ_e_’) refers to an elementwise Hadamard product. E_i_ refers to specific environment ‘i’ and is also used in ‘Z_ei_’ to denote block-diagonal matrices to model environment-specific variance components. ‘E’ in all the models correspond to environment group factor that is modelled as a fixed effect.
