# Supplementary material for: GS4PB: An R Shiny application to facilitate a genomic selection pipeline for plant breeding
Source: Plant Genome. 2025 Dec 11;18(4):e70150. doi: 10.1002/tpg2.70150 (PMC12698896; doi:10.1002/tpg2.70150)
Supplement: Supplementary file 1 — Supplementary Material [file TPG2-18-e70150-s003.docx]

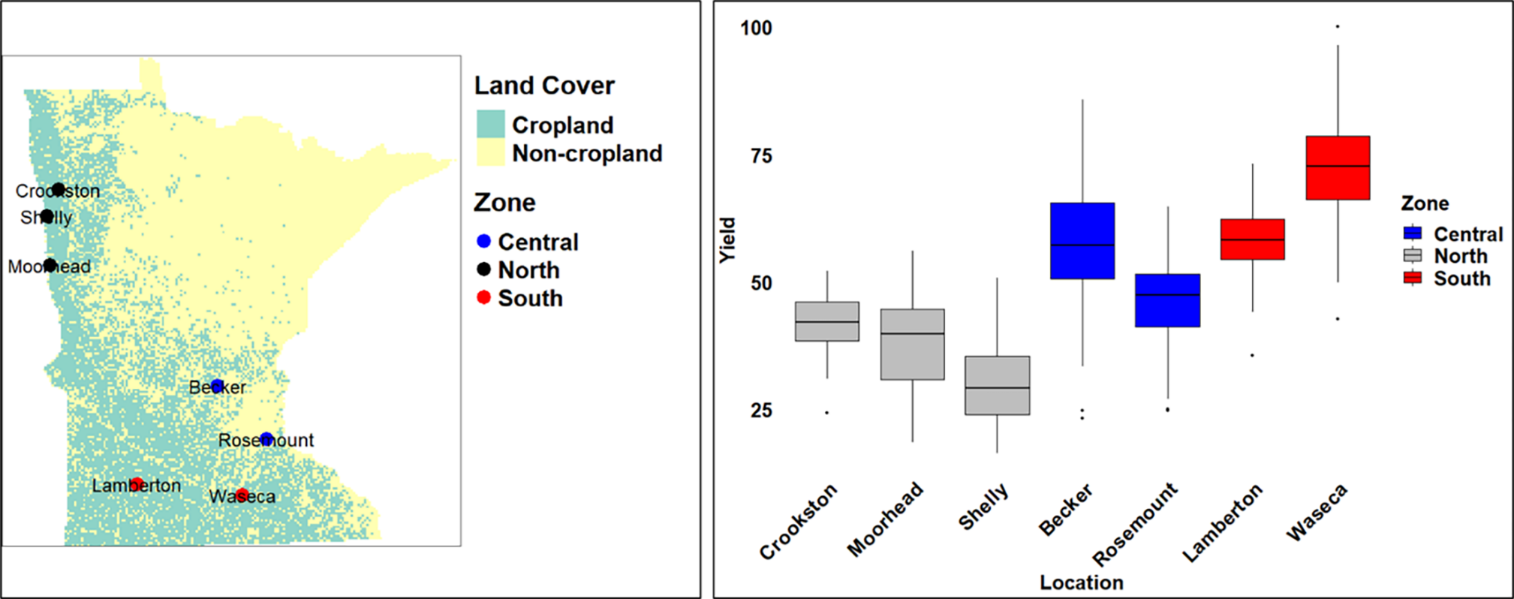


**Supplementary Figure 1.** Left panel: 2023 Preliminary Yield Trial (PYT) locations in Minnesota, USA. Crookston, Moorhead and Shelly represent the northern zone (black); Becker and Rosemount represent the central zone (blue); and Lamberton and Waseca represent the southern zone (red) in Minnesota for the PYTs conducted in 2023. Right panel: Distribution of 2023 PYT best linear unbiased estimates of breeding lines effects for yield across the northern (gray), central (blue) and southern (red) zone locations.


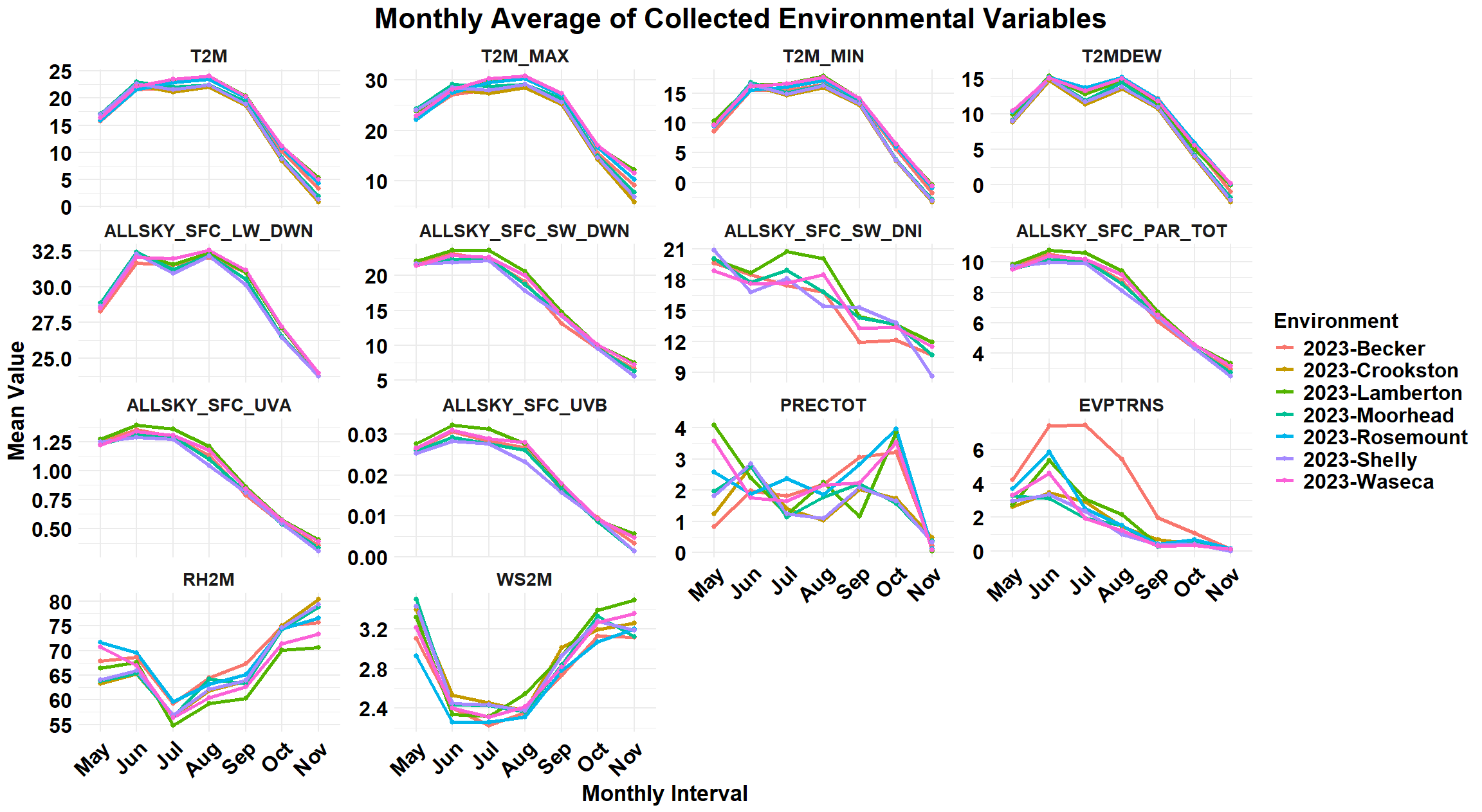


**Supplementary Figure 2.** Monthly Average of environmental covariables collected from NASA POWER database for each of the 2023 PYT locations (see color legend for specific locations). Daily data for “T2M“, "T2M_MAX”, "T2M_MIN“, "T2MDEW”, "ALLSKY_SFC_LW_DWN“, "ALLSKY_SFC_SW_DWN", , "ALLSKY_SFC_SW_DNI", "ALLSKY_SFC_PAR_TOT", "ALLSKY_SFC_PAR_TOT", "ALLSKY_SFC_PAR_UVA", "ALLSKY_SFC_PAR_UVB”, "PRECTOT”, "WS2M“, "RH2M”, and “EVPTRNS” were extracted from NASA power database for the period ranging from 1-May-2023 to 30-Nov-2023 and monthly averages were estimated for each of the covariates using the “summarizeWTH” function in *EnvRtype* package. Detailed descriptions of each environmental covariable are provided in Supplementary Table 2.


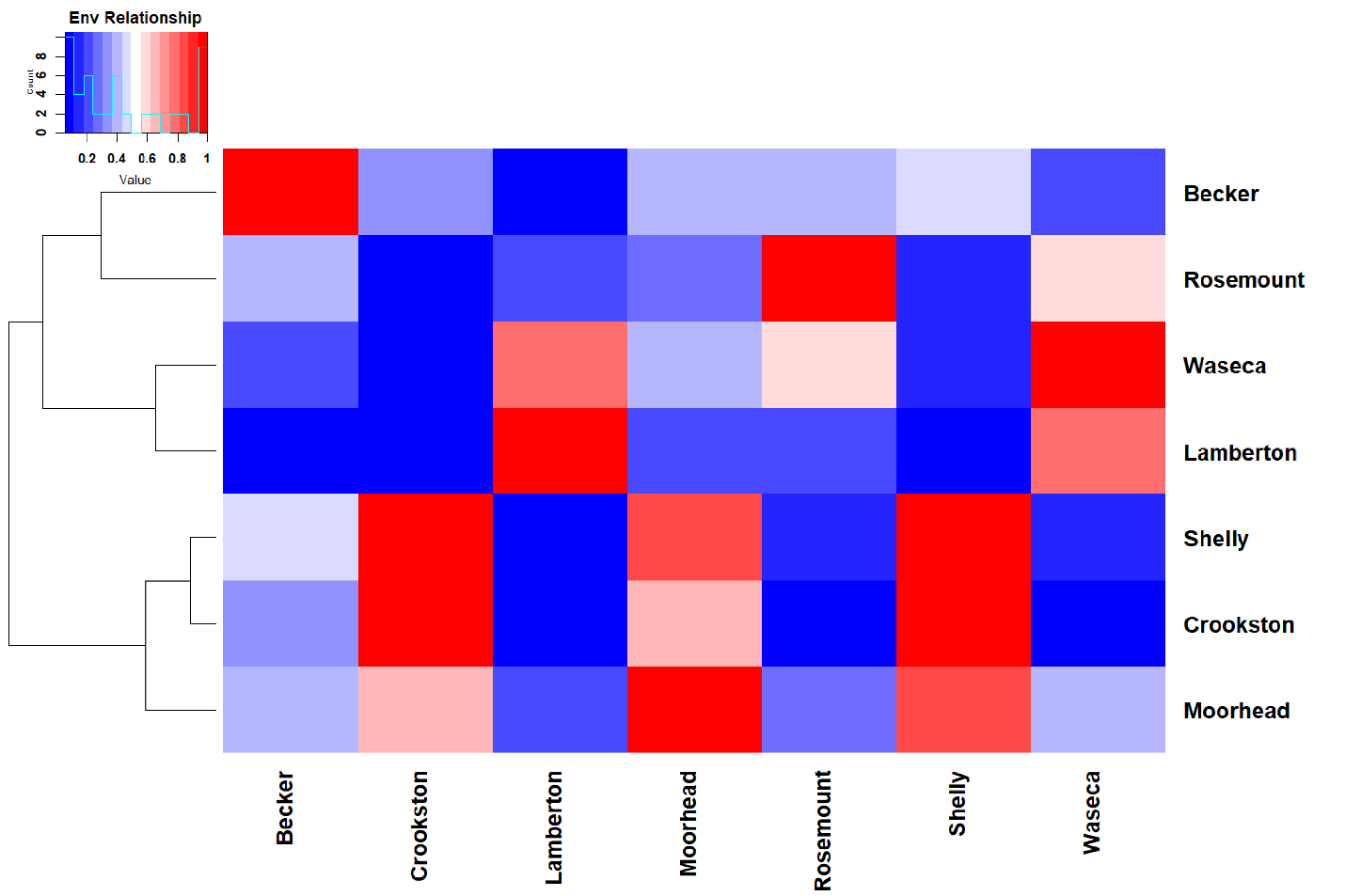


**Supplementary Figure 3.** Heatmap of “environmental kinship” estimated with a Gaussian kernel using the ‘getKernel’ function in *BGGE/EnvRtype* package. All the environmental variables plotted in Supplementary Figure 2 were used for the estimation of environmental kinship. The dendrogram is based on Euclidean distance between row means.


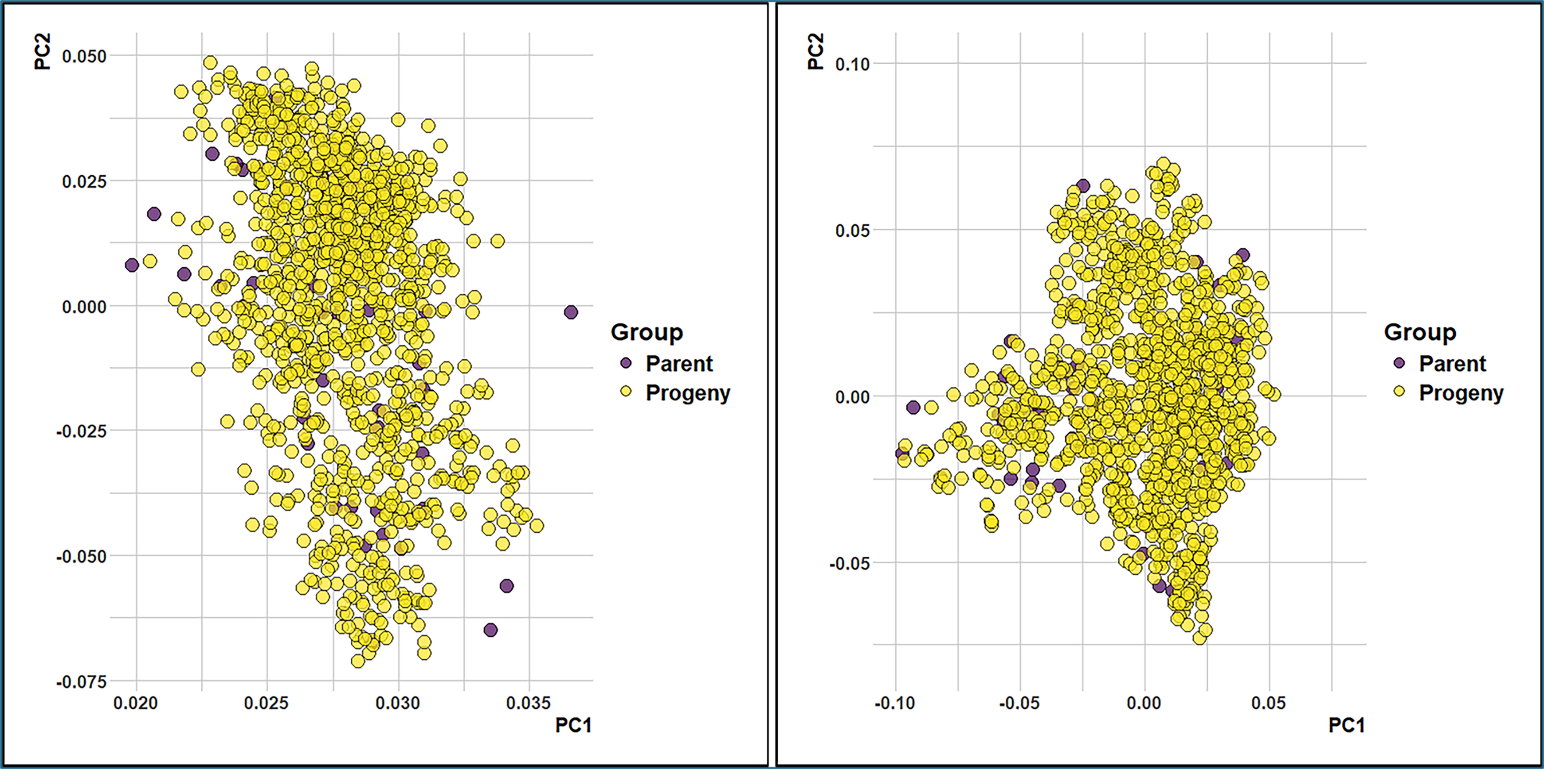


**Supplementary Figure 4.** Principal component analysis of genotypic data of 2023 PYT breeding lines (yellow) and their parents (purple). The genotypic data from Agriplex Soy 1K SNP assay (left panel) and Agriplex Soy 1K SNP assay data imputed up to 32.8K SNPs of the SoySNP50K set using the parental lines as reference (right panel). Loading score correlations of 0.842 and 0.836 were observed for PC1 and PC2, respectively, between the 1K SNP set and the 1K set imputed up to the 32.8K SNP data sets. The loading scores represent the relative contribution of genotypes to variation in the two major principal components in both the datasets. In addition, a correlation of 0.849 was observed between the kinship matrices estimated using the VanRaden method for the 1K set and the 1K set imputed up to 32.8K SNP set.
