# Supplementary material for: GS4PB: An R Shiny application to facilitate a genomic selection pipeline for plant breeding
Source: Plant Genome. 2025 Dec 11;18(4):e70150. doi: 10.1002/tpg2.70150 (PMC12698896; doi:10.1002/tpg2.70150)
Supplement: Supplementary file 2 — Supplementary Material [file TPG2-18-e70150-s004.docx]

**Supplementary Table 1.** Performance profiling results (memory allocated in MegaBytes (MB) / CPU time in minutes) obtained using ‘Rprof’ for three pipelines using three genotypic data sets of varying sizes. Total memory and total time were estimated using ‘summaryRprof’. Profiling was performed on an interactive Linux desktop system with 32 cores, 500 GB memory and 190 GB memory scratch at MSI (Minnesota Supercomputing Institute at UMN).

| **Pipeline** | **Steps in the pipeline** | **Marker number / Population size** | | |
| --- | --- | --- | --- | --- |
|  |  | **1.2K / 2736** | **5.2K / 2896** | **39K / 2890** |
|  |  | **Memory Allocated (MegaBytes) / CPU Time** | | |
| Single Trait (ST) in Single Environment | Load Geno-Filter Geno-Impute LDKNNI-Load Pheno-Merge Data-ST Cross validation  (10-fold / 10-rep) -ST Genomic Prediction | 8752.5 MB /  1.6 min | 31,812.9 MB /  5.6 min | 199,057.4 MB / 55.8 min |
| Multiple Traits (MT) in Single Environment | Load Geno-Filter Geno-Impute LDKNNI-Load Pheno-Merge Data-MT Cross validation  (10-fold / 10-rep) -MT Genomic Prediction | 12,611.1 MB /  1.8 min | 53,484.2 MB /  5.7 min | 292,154.1 MB / 63.4 min |
|  | | **Marker number / Population size** | | |
|  |  | **1.2K / 1271** | **4.3K / 1244** | **32K / 1244** |
| Single Trait in Multiple Environments (ME) | Load Geno-Filter Geno-Impute LDKNNI-Load Pheno-Merge Data ME Cross validation 1  (10-fold / 10-rep)-ME Genomic Prediction | 13,903.4 MB /  2.2 min | 10,960.8 MB /  3.4 min | 97,439.9 MB /  48.9 min |

Note: User time in (mins) may be higher than CPU time from ‘Rprof’ depending on the system and dynamic allocation of resources. For this profiling trial, cross validation steps were implemented using 10 cores. For single environment GS pipelines, 1.2K data refers to a genotypes table with 2736 strains and 1205 markers; 5.2K data refers to a genotype table with 2896 strains and 5245 markers; 39K data refers to a genotype table with 2890 strains and 39,382 markers. For multi-environmental GS pipelines, 1.2K data refers to a genotype table with 1271 strains and 1220 markers; 4.3K data refers to 1244 strains and 4285 markers; 32K data refers to 1244 strains and 32021 markers. Imputation using LDKNNI method and cross validation were the most time-consuming steps in the pipeline for large genotypic tables, which highlights the potential for further optimization of these methods to improve processing speed for this GS pipeline implementation.
